# Psychosocial Functioning of Individuals at Risk of Developing Compulsive Buying Disorder

**DOI:** 10.3390/jcm13051339

**Published:** 2024-02-27

**Authors:** Kamila Rachubińska, Anna Maria Cybulska, Aleksandra Szylińska, Ewa Kupcewicz, Dorota Ćwiek, Ireneusz Walaszek, Elżbieta Grochans

**Affiliations:** 1 Department of Nursing, Faculty of Health Sciences, Pomeranian Medical University, Żołnierska 48, 71-210 Szczecin, Poland; kamila.rachubinska@pum.edu.pl (K.R.); ireneusz.walaszek@pum.edu.pl (I.W.); elzbieta.grochans@pum.edu.pl (E.G.); 2 Department of Cardiac Surgery, Pomeranian Medical University, Powstańców Wlkp 72, 70-111 Szczecin, Poland; aleksandra.szylinska@gmail.com; 3 Department of Nursing, Collegium Medicum, University of Warmia and Mazury in Olsztyn, 10-719 Olsztyn, Poland; ekupcewicz@wp.pl; 4 Department of Obstetrics and Pathology of Pregnancy, Pomeranian Medical University in Szczecin, ul. Żołnierska 48, 71-210 Szczecin, Poland; dorota.cwiek@pum.edu.pl

**Keywords:** behavioural addiction, compulsive buying, depression, eating disorder, personality traits, neuroticism, workaholism

## Abstract

(1) **Background:** This study aimed to establish the connection between depressiveness, workaholism, eating disorders, and personality traits, according to the five-point model called the Big Five, in women with a risk of compulsive buying disorder. (2) **Methods:** The study was conducted on 556 Polish women from the West Pomeranian Voivodeship. The study employed the diagnostic survey method using a questionnaire technique including Personality Inventory NEO-FFI, the Buying Behaviour Scale, the Beck Depression Inventory I-II, the Three-Factor Eating Questionnaire, and a self-questionnaire. (3) **Results:** The analysis revealed the risk of compulsive buying being accompanied by a higher median score for depressiveness, neuroticism, Cognitive Restraint of Eating, Uncontrolled Eating, and a risk of workaholism. A lower score in the respondents in the compulsive buying risk group was observed in an assessment of agreeableness and conscientiousness. Work addiction was exhibited by 26% of people with compulsive buying disorder vs. 12% of people without it. (4) **Conclusion:** This study found that a high risk of compulsive buying disorder is accompanied by a high risk of moderate depressiveness, neuroticism, Cognitive Restraint of Eating, Uncontrolled Eating, and workaholism. It also confirmed the view that compulsive buying is a behavioural addiction which is a consequence of ineffective coping and being dissatisfied with one’s social life.

## 1. Introduction

Technological progress, exposure to positive stimuli, and disregard for emotional control and self-awareness in the process of personality formation make it difficult for individuals to control their behaviour. Such loss of control results in addiction development [1].

Very recently, compulsive buying disorders have been linked to disorders resulting from addictive behaviour because of phenomenological and potential neurocognitive similarities. If a behaviour is considered addictive, (neuroscientific) theories should be applied to the phenomenon. Otherwise, it would be unjustified to call the phenomenon an addiction but rather an impulse control disorder or obsessive compulsive disorder [2]. Current theories that are considered particularly relevant to the study of addictions, both to psychoactive and behavioural substances, include incentive–sensitisation theory [3]; the response inhibition and salience attribution model (iRISA) [4]; reward deficiency syndrome; dual-process approaches of addiction [5], including those focusing on latent cognition [6]; and more specific models of behavioural addiction. The latter group includes models such as the cognitive-behavioural disorder model [7], the triadic disorder model [8], and the interaction model of Person–Affect–Cognition–Execution (I-PACE) model [9]. According to the I-PACE model, the urge to buy can be triggered by internal factors (e.g., discomfort, boredom, self-doubt) and/or external factors (e.g., advertising, seeing influencer posts, having extra money). Repeatedly experiencing positive feelings or relief from negative mood states while shopping can result in an attentional bias related to these triggers, which, in turn, can reinforce the need to buy (at a later stage, the hunger to buy) and lead to an increase in purchase activity. These interactions may be moderated by reduced general inhibitory control in the early stages and mediated by stimulus-specific inhibitory control deficits in the later stages of compulsive buying disorders, which may result in increasing habitual maladaptive buying patterns [10,11]. Affective and cognitive processes are likely to be associated with neuroadaptive changes in frontal–striatal circuits over time [10,12].

As with other psychiatric disorders, there is no doubt that clarifying the diagnostic criteria for compulsive buying disorders is important for making appropriate health policies [13]. One methodological approach that has been applied to other areas of diagnostic uncertainty is the Delphi process. This approach was originally known as a forecasting method and has been applied to many other areas, including mental health [14]. The method uses a systematic sequence of repeated rounds of voting to establish expert consensus on a problem for which precise information is lacking [15,16]. A growing number of experimental studies indicating that CBSD falls within the ICD-11 category of “other specified disorders due to addictive behaviours” [2,17,18] are being published, yet this requires further evidence-based discussion [19,20].

Compulsive buying is defined as excessive or poorly controlled impulses, being absorbed by something or behaviours that involve shopping, and spending money, which result in negative effects. These behaviours are motivated more by the urge to get rid of and escape from negative emotions than by the need to spend money and by materialism [21,22]. Impulsive buying is associated with an irresistible desire to buy and a sudden decrease in tension after the purchase. Buyers not only perform repeated acts of problematic buying but also have undesired, recurring thoughts of buying, which gives one an impression of an obsessive compulsive disorder [23]. A purchase gives one a momentary relief, which is ultimately replaced by a feeling of guilt and disappointment and can result in debt, relationship issues, an increased risk of criminal behaviour, or even suicide attempts [24,25]. 

It is estimated that compulsive buying disorder affects 1% to 10% of the adult population in many countries, including France, Spain, Canada, Germany, and Australia [26]. According to a 2015 study, it may also affect 4.1% of the population of Poland aged 15 years or more [27].

The aetiology of compulsive buying disorder is affected by diverse biological, psychological, and cultural factors and the coexistence of other addictions, e.g., workaholism. Sex is another predictor for compulsive buying. Women account for approx. 80% of those affected by compulsive buying disorder [28,29]. These inclinations arise not only from socio-cultural changes and an increase in the general wealth of societies but also depend on individual characteristics. The causes of excessive buying are believed to include the role of subjective properties, especially the presence of depressiveness symptoms [30,31]. Studies conducted by McElroy et al. [32] provided data suggesting that 95% of compulsive buyers suffer from mood disorders, 80% from anxiety disorders, and nearly half of them exhibited impulse control disorders. Lejoyeux et al. [33] mentioned the presence of depression symptoms in compulsive buyers. Such individuals treat this activity as a way of alleviating unpleasant emotions. Therefore, one can assume that for D-type individuals who typically experience negative emotions and difficulties in expressing them, going shopping would be a good idea to cope with them [34]. 

Personality-related correlates are significant factors associated with compulsive buying. The Big Five personality traits can be a risk factor or a protective factor for addiction [35,36]. It has been demonstrated in many studies that neuroticism and extraversion are positively associated with compulsive buying [37,38]. On the other hand, a negative correlation was demonstrated between conscientiousness and compulsive buying [39]. Conscientiousness may be a protective factor against compulsive buying because individuals with a low level of this trait are more prone to be less organised, irresponsible, and independent [40,41,42,43].

The literature indicates that a high risk of compulsive buying disorder in women is accompanied by a high risk of moderate depressiveness, Cognitive Restraint of Eating, Uncontrolled Eating, and workaholism [44]. 

It is necessary to take up further studies in this regard. Moreover, this study aimed to establish the connection between depressiveness, workaholism, eating disorders, and personality traits, according to the five-point model called the Big Five, in women with a risk of compulsive buying disorder.

## 2. Materials and Methods

### 2.1. Settings and Design

The respondents included 556 women from the West Pomeranian Voivodship in Poland. The choice of the study sample was made with the use of a sample size calculator in the STATISTICA programme for Windows 13.1 (TIBCO Software Inc., StatSoft, Poland) with a 95% confidence interval. This constituted a representative sample. Based on the number of women in the Voivodship of West Pomerania, the minimum number of patients that could have been included in the study is 384 people. The group was selected at random. No randomisation tool was applied. Female volunteer participants who met the enrolment criteria were enrolled. The following were the inclusion criteria: female sex, age ≥ 18 years, place of residence in the Voivodship of West Pomerania, no self-reported mental illnesses, signing an informed consent for participation in the study, and completing the questionnaires. The study was approved by the Bioethics Committee at the Pomeranian Medical University in Szczecin (KB-0012/518/12/16) and conducted in compliance with the Declaration of Helsinki. After the study was approved, the study questionnaires prepared earlier were given by trained pollsters to women, who read the information and consented to their participation in the project. The participants were informed about the aim of the study, and they were able to ask questions and were given exhaustive answers. The study was conducted using the traditional method of distributing paper copies of the questionnaires. Altogether, 605 women were invited to take part in the study. Only 556 women completed the questionnaire correctly (return rate: 92%). The patients were divided in accordance with this result based on the Buying Behaviour Scale. Respondents with a score under 44 points were categorised as being in the “norm” group, and those with a score above or equal to 44 points were categorised as high risk /tendency for compulsive buying disorder. This study is part of a larger project concerning the incidence of behavioural addictions in women [45,46].

### 2.2. Surveys

The study was conducted via a diagnostic survey using the questionnaire technique. Standardised tools, adapted to the Polish conditions, were used.

#### 2.2.1. Personality Inventory NEO-FFI

Personality Inventory NEO-FFI is a standardised tool used to analyse the personality traits comprising the five-factor model proposed by Costa and McCrae, known as the Big Five. It is divided into five subscales that measure neuroticism, extraversion, openness to experience, agreeableness, and conscientiousness. Each subscale contains 12 statements, which are given scores from 0 to 4 by the study participants. The scoring direction is reversed in some cases. Possible raw scores lie within the range from 0 to 48 points and are converted to stens. The higher the result in a subscale, the larger the attribute intensity. The raw scores are converted into sten scores and expressed by five factors: neuroticism (N) (Cronbach’s alpha 0.890), extraversion (E) (Cronbach’s alpha 0.807), openness to experience (O) (Cronbach’s alpha 0.798), agreeableness (A) (Cronbach’s alpha 0.763), and conscientiousness (C) (Cronbach’s alpha 0.855). The obtained value of Cronbach’s α statistics was 0.924 [47,48].

#### 2.2.2. Buying Behaviour Scale (BBS)

The BBS allows for one to determine the overall outcome of buying behaviour and its two factors, i.e., compulsion and lack of control, as well as the alleviation of tension and negative emotions. This tool comprises 16 statements graded on a 5-point scale. Total scores range from 16 to 80 points. The higher the score, the greater the tendency for compulsive buying. A higher tendency for compulsive buying is indicated by a score of more than 44, and a lower tendency is indicated by a score of under 35 points. Scores lying between 35 and 44 points are indicative of a moderate tendency for compulsive buying. The internal consistency of the questionnaire was assessed with the Cronbach alpha index, which was 0.92 [49]. 

#### 2.2.3. Beck Depression Inventory—BDI I-II

The Beck Depression Inventory (BDI I-II) is used in the self-assessment of depressive disorder intensity. It contains 21 questions with 4 answer options. The final score is calculated by adding up the points for each question. The score reflects the depression level and is interpreted in the following manner: 0–13—no or minimum depression symptoms, 14–19—mild depression, 20–28—moderate depression, and 29–63—severe depression. The BDI-II demonstrated adequate internal consistency (α = 0.87) [50].

#### 2.2.4. The Work Addiction Risk Test (WART) 

This is a questionnaire measuring the symptoms of a workaholic behaviour pattern. This tool comprises 25 items that measure behavioural, cognitive, and emotional responses which are regarded as workaholism syndrome. The 25 statements are assessed on a four-point scale of the frequency of workaholism symptoms. This questionnaire measures fully developed workaholism syndrome or the risk of work addiction, depending on the score. Scores range from 25 to 100 points. The effect of work compulsion is indicated by a score of above 56 points. A high score (67–100 points) indicates a strong addiction, and a moderate score (57–66 points) indicates moderate work addiction. A low score, within the range of 25–56 points, indicates the absence of addiction and a degree of addiction to work (the higher the score, the higher the probability of workaholism development) [51,52,53].

#### 2.2.5. The Three-Factor Eating Questionnaire (TFEQ-13)

The questions contained in the TFEQ-13 questionnaire are indexed in three subscales that measure cognitive, behavioural, and emotional aspects of eating behaviour. The questions in the TFEQ-13 questionnaire comprise three factors: (1) Cognitive Restraint of Eating, (2) Uncontrolled Eating, and (3) Emotional Eating. The first subscale measures behaviour associated with placing restrictions on the amount or type of food consumed in order to control one’s body weight and the image of one’s body. The second subscale measures a tendency to eat more than usual as a result of loss of control over eating or uncontrolled hunger, which causes compulsive eating. The third subscale measures episodes of excessive eating caused by a bad mood and anxiety. These three factors reproduce 56.8% of the variation in the whole set of variables observed in the experiment. The internal consistency index—Cronbach’s alpha—was 0.78 for the entire scale, and for the subscales, the following values were found: 0.78, 0.76, and 0.72. The TFEQ-13 questionnaire contains standardised answers on a 4-point scale from 0 to 3 (definitely yes—3, rather yes—2, rather not—1, definitely not—0). Question 13 (R5) was transcoded in the following manner: 1 and 2—0; 3 and 4—1; 5 and 6—2; 7 and 8—3. The values are calculated separately for each subscale. No values are calculated for the whole scale. A higher total score in a subscale indicates an intensified disorder lying within its range [54,55].

#### 2.2.6. Self-Questionnaire

The self-questionnaire contained close-ended and semi-open-ended questions aimed at acquiring selected demographic data for the participants, including data on age, educational background, marital status, place of residence, and professional activity.

### 2.3. Statistical Analysis 

Our statistical analysis was performed with the licenced Statistica 13.0 program (StatSoft, Inc., Tulsa, OK, USA). The group characteristics were provided with descriptive statistics, mainly the mean and median values, standard deviations, and counts and percentages. The Shapiro–Wilk test was employed to assess the variable distribution normality in the study. The Mann–Whitney U test was employed to analyse data for the two groups. Qualitative data were analysed with the X2 test. For subgroups with a small number of elements, Yates correction or Fisher’s exact test was applied. Logistic regression was performed, and the results are presented as odds ratios (ORs) with a 95% confidence interval. A multivariate analysis adjusted by age was performed. Akaike’s Information Criterion (AIC) was applied to determine which model fits best. A level of significance of *p* ≤ 0.05 was adopted.

## 3. Results

### Brief Characteristics of the Respondents

A total of 556 women residing in the West Pomeranian Voivodeship participated in the study. Slightly more than half of the participants had pursued higher education (51.62%) and resided in a locality with a population of more than 100,000 inhabitants (52.70%). Most women reported being in a non-formal relationship (66.55%). A detailed description of the participants’ characteristics is provided in Table 1. 

The patients were classified according to the results based on the buying behaviour scale. An assessment of the selected scales with respect to the risk of compulsive buying is shown in Table 2. The analysis revealed the risk of compulsive buying being accompanied by a higher median score for depressiveness according to the BDI I-II (*p* = 0.021), neuroticism according to the NEO-FFI (*p* = 0.003), Cognitive Restraint of Eating according to the TFEQ-13 (*p* = 0.004), Uncontrolled Eating according to the TFEQ-13 (*p* = 0.019), and a risk of workaholism according to the WART (*p* < 0.001). A lower score in the respondents in the compulsive buying risk group was observed in an assessment of agreeableness according to the NEO-FFI (*p* < 0.001) and conscientiousness according to the NEO-FFI (*p* = 0.028). Work addiction was exhibited by 19 (26%) people with compulsive buying disorder vs. 60 (12%) people without it (*p* < 0.001) (Table 2).

An assessment of the relationship between selected scales and the risk of compulsive buying is shown in Table 3. A multivariate analysis revealed a higher risk on the BDI I-II scale (OR = 1.052, *p* = 0.001) of neuroticism (OR = 1.034, *p* = 0.012), Cognitive Restraint of Eating (OR = 1.122, *p* = 0.009), Uncontrolled Eating (OR = 1.103, *p* = 0.035), WART (OR = 1.050, *p* < 0.001), and addiction to work (OR = 2.384, *p* = 0.004) among patients with compulsive buying disorder. After analysing multiple models, the age-adjusted WART model was shown to be the best model.

## 4. Discussion

Changes taking place in the contemporary world have a considerable impact on human life and create favourable conditions for the development of addictions. Therapists, doctors, and researchers are being increasingly faced with cases of compulsive behaviour focused on a specific activity. Apart from addictive behaviours such as gambling or playing computer or web games, some people are also absorbed by shopping (compulsive buying), sexual activity, or work (workaholism). A shopping addiction is understood as compulsive, excessive buying and spending money, which results in negative consequences. The main contribution of this study lies in its identification of the basic psychological factors associated with the risk of compulsive buying [40,56]. Its findings provide the following contributions to the literature. 

Shopping addiction (compulsive buying disorder) has been attracting increasing interest recently. It can have an adverse impact on individuals’ social and professional life, as well as their families. This addiction is associated with a high rate of concomitant mental diseases. Affective disorders are mental diseases most frequently reported in compulsive buyers. Mueller et al. [57] reported that the incidence of any affective disorders throughout one’s life is 68% in individuals with compulsive buying disorder. Depression is the most frequently diagnosed affective disorder in this population [44,58,59].

Mueller et al. [57] determined the incidence of compulsive buying disorder to be 6.9%. In their study, addicts were more depressive than non-addicts. Otero-López and Villardefrancos [60] demonstrated the incidence of compulsive buying disorder to be 7.1%. Female sex, depressiveness, anxiety, and young age were predictors for compulsive buying. Many studies have shown its higher prevalence in women than in men [44,56,57,58,59,60,61,62].

Mental disorders such as depression can increase the risk of addiction [57,58], and they can lead to addiction and vice versa [63,64,65,66,67]. Lejoyeux et al. [33] studied the incidence of compulsive buying disorder among hospitalised patients with a severe depressive episode. Young age, female sex, and being single were the most frequent characteristics of the addicts. Compulsive buyers exhibited more depression symptoms, as assessed by the German version of the Brief Patient Health Questionnaire Mood Scale (PHQ-9). Black et al. [1] demonstrated that compulsive buyers experience mood disorders, anxiety, depressiveness symptoms, and ADHD throughout their lives. According to the findings of a study by Suresh et al. [68], psychological factors, i.e., loneliness, depression, low self-esteem and anxiety, are positively associated with Internet addiction and compulsive buying online. 

Compulsive buying leads to serious consequences for an individual, and these consequences may have a great impact on the individual’s environment and society. Depression and low self-esteem may have an influence on a compulsive buyer and their personal relations [57]. They can also lead to huge debts (58.3%), not being able to pay the bills (41.7%), criticism from friends (33.3%), and penal and financial consequences (8.3%) [69]. Obviously, compulsive buying affects all individuals in society [69,70,71]. Dittmar et al. [44] claimed that shopping could be a form of self-treatment for “negative emotions” such as depressive symptoms, since it can modify one’s mood and give one pleasure, excitement, and a sense of being in control. 

A risk of addiction to work was observed in 26% of the women in this study at risk of compulsive buying, whereas a risk of work addiction was observed in only 12% of those without such a risk. Similar to compulsive buying, work addiction is defined as an obsessive compulsive disorder. Spending long hours at work to satisfy one’s financial needs does not make one a workaholic. Addicts are driven by an internal need. Work satisfies their “mental hunger”. “I work, therefore I am”—this phrase illustrates their reasoning. Work defines their identity, makes their life meaningful, and helps them gain approval and acceptance. Like buying new things, work becomes the only method for proving their value while at the same time reducing the discomfort arising from unfulfilled needs. Workaholics believe that they can gain other people’s respect only if they work hard [72]. Perfectionism and reliability are important factors which favour one’s addiction to work. According to the findings of Polish studies, a good financial situation favours the development of buying issues [26].

It was shown in an American study that the percentage of those addicted to shopping ranged from 1.8% to 16%. The majority of those addicted (nearly 80%) were women. These inclinations arise not only from sociocultural changes and an increase in the wealth of a society but also depend on individual characteristics. Studies conducted by McElroy et al. [32] provided data suggesting that 95% of compulsive buyers suffer from mood disorders, 80% suffer from anxiety disorders, and nearly half of them exhibit impulse control disorders. Lejoyeux et al. [33] mentioned the presence of depression symptoms in compulsive buyers. Such individuals treat this activity as a way of alleviating unpleasant emotions. Therefore, one can assume that for D-type individuals who typically experience negative emotions and difficulties in experiencing them, shopping would be a good idea to cope with them. This study aimed to determine the relationship between a D-type personality and the risk of compulsive buying. On the other hand, workaholics find it difficult to handle emotions and cope with them. Workaholics also experience interpersonal issues. These arise mainly from putting work ahead of other forms of spending one’s time, including maintaining contact with other people, as well as a growing distance towards others, which is a consequence of being excessively absorbed with work [73]. A special role in the development of addiction to eating can be played by emotions and stress associated with them [74]. This is because eating is a way of relieving tension that is simple, easily available, and has an immediate effect. It also helps one to distract one’s attention from stressful stimuli. An individual with a D-type personality seems to be predisposed to escaping into excessive and uncontrolled eating (compulsive eating disorder), mainly because of a tendency for negative emotions but also because of difficulties coping with them. Workaholics, individuals with compulsive buying and/or compulsive eating disorders, and those with a D-type personality exhibit some common characteristics, which include experiencing negative emotions (especially anxiety) and interpersonal relationship issues. Therefore, it can be expected that there is a significant relationship between this personality type and excessive involvement in work and compulsive buying [34].

There is scarce data on the incidence of compulsive buying disorder and even less data related to the personality factors affecting those types of behaviour [34]. According to this study, women with a great tendency for compulsive buying exhibit a neurotic personality. This study also suggests that personality traits can play a key role in some people’s susceptibility to the development of compulsive buying. The Big Five model, which is the most common perspective of human personality structure examination, suggests the existence of five basic personality traits (neuroticism, extraversion, openness, agreeableness, and conscientiousness) [75]. There are various methods of handling compulsive buying based on the traits under analysis. The findings of earlier studies, regardless of the type of sample used, are highly consistent with respect to the claim that neuroticism (a tendency for experiencing negative emotions such as anxiety and depression) is a risk factor [76]. Highly neurotic individuals tend to be unable to control impulses, and they are self-aware, meaning that online shopping becomes a market both for impulsive buying and for avoiding social interactions [77]. According to the available literature, conscientiousness (a tendency for self-discipline) is a protective agent against compulsive buying. It has been shown in many studies that a low level of conscientiousness can be a risk factor for compulsive buying [60,78]. Faber and Vosh [79] claim that “compulsive buying is a form of failure of self-regulation”. Additional proof of the importance of this trait in various aspects (money control, debt, behavioural addiction) comes from many studies. 

With regard to the link between agreeableness (a tendency for altruism, trust, modesty and cooperation) and compulsive buying, some studies suggest a positive relationship, while according to others, the relationship is negative [80,81]. However, when it comes to openness (a tendency for curiosity and creativity and preferring novelty and diversity) and extraversion (a tendency for being sociable, warm, active, assertive, good-natured, and stimulation-seeking), the findings are still inconclusive [82]. Those who score high in openness are unconventional and open to new experiences, which may lead them to explore shopping. High scores in extraversion are indicative of a person who is sociable and prefers activity and a quick pace of life [77]. 

A comparison between compulsive and non-compulsive buyers in the Big Five personality domains showed that the group of compulsive buyers scored higher on the neuroticism subscale. These findings are consistent with those from some earlier studies conducted on both general population samples [77] and clinical ones [83], which link emotional instability with compulsive buying. The proof for this pattern of results also comes from recent research which emphasises the role of neuroticism and conscientiousness in other addictions such as compulsive eating [84], Facebook addiction [85], Internet addiction, compulsive smartphone use [86], and problematic gambling [87]. 

According to Baumeister’s escape theory [88], buying behaviour can be understood as an attempt to escape from negative feelings by focusing on external stimuli such as buying. The widely accepted definition of compulsive buying, proposed by O’Guinn and Faber [8], also refers to the fact that individuals become involved in compulsive buying as a way of relieving negative feelings. Many research studies have characterised compulsive buyers as emotionally unstable individuals who experience negative emotions such as anxiety or depression to a greater extent than non-compulsive buyers [83,89] and also as more impulsive ones [90,91].

The prevalence of compulsive buying has been increasing over the past few decades, and it is becoming typical of modern consumer societies. It is conceptualised as chronic and repeated shopping becoming the main response to negative events or feelings, which provides short-term rewards but results in negative long-term consequences, both personal and family-related [17]. 

### Limitations and Recommendations for Further Research

The findings of this study can be used to propose certain implications for professional practice. The main asset of this study was that it employed standardised tools adapted to Polish conditions, which considerably enriched the presented data. It could be an important goal for therapists working with compulsive buyers to develop educational guidelines to minimise the risk of the compulsive buying disorder associated with some personality traits. Given the direct impact of neuroticism on this behaviour pattern, it seems reasonable to design interventions aimed at reducing the impact of this personality trait and depressiveness on excessive buying, which could be useful in the treatment and prevention of issues associated with excessive buying. This study also had certain limitations which we hope will be overcome by future research. Firstly, the cross-sectional, correlational nature of this project limits the ability to identify causal relationships. The absence of data on the risk of psychosocial functioning disorders could be of importance for the differentiation of the impact of variables on the occurrence of the risk of compulsive buying. Various socio-cultural aspects and other interesting variables (e.g., level of income, socioeconomic status, or social class) are only some of the aspects that should be considered in future research. Problems with the generalisation of data to other cultures are another limitation. Our risk assessment for compulsive shopping and related factors was based only on the measurement of self-descriptive constructs. Information from autobiographical narratives may be necessary to obtain the whole picture of an individual’s behaviour. An assessment based on self-descriptive tools may be distorted because one wants to be perceived as attractive, i.e., with a tendency to avoid criticism and provide more socially acceptable answers. This may result in overestimating health-promoting behaviours and underestimating undesirable ones. Participants were provided with information about the study aims, and this potentially may have affected the responses provided by the participants.

Despite its limitations, this study has provided important conclusions and can be a starting point for more extensive research aimed at identifying a relationship between depressiveness, the personality traits comprising the Big Five model, and the risk of compulsive buying disorder occurrence among Polish women. New studies could integrate personality traits, characteristic adaptations, and life history data associated with excessive buying. Using larger samples from various socio-cultural contexts and noting other interesting variables (e.g., level of income or social class) are only some of the aspects that should be considered in the future. Research may help to overcome this weakness and better understand the complex nature of excessive buying. Despite this study’s limitations, its findings lead one to an interesting scenario which suggests that material values—from intervention to prevention—can be a potentially useful goal in reducing the negative impact of some traits on excessive buying.

In this context, the knowledge of common and specific risk factors associated with compulsive buying could allow for the development of better programmes for early prevention and intervention.

## 5. Conclusions

This study has shown that a high risk of compulsive buying disorder is accompanied by a high risk of moderate depressiveness, neuroticism, Cognitive Restraint of Eating, Uncontrolled Eating, and workaholism. After analysing multiple models, the age-adjusted WART model was shown to be the best model. It has also confirmed the view that compulsive buying is a behavioural addiction which is a consequence of ineffective coping and being dissatisfied with one’s social life.

## Figures and Tables

**Table 1:** General sociodemographic characteristics of the study group (N = 556).

		Total (n = 556)	Norm (n = 483)	Risk of the Compulsive Buying Disorder (n = 73)	*p*
Age [years], Me (Q1–Q3)		27.5 (22.0–45.0)	29 (22.0–46.0)	25.0 (22.0–33.0)	0.038
Educational background, n (%)	University	287 (51.62%)	250 (51.76%)	37 (50.68%)	0.452
	Secondary	254 (45.68%)	222 (45.96%)	32 (43.84%)	
	Vocational	3 (0.54%)	2 (0.41%)	1 (1.37%)	
	Elementary	12 (2.16%)	9 (1.86%)	3 (4.11%)	
Place of residence, n (%)	Town, population > 100 thousand	293 (52.70%)	256 (53.0%)	37 (50.7%)	0.807
	Town, population 10–100 thousand	249 (44.78%)	216 (44.72%)	33 (45.21%)	
	Town, population under 10 thousand	4 (0.72%)	3 (0.62%)	1 (1.37%)	
	Village	10 (1.80%)	8 (1.66%)	2 (2.74%)	
Marital status, n (%)	Non-formal relationship	370 (66.55%)	327 (67.70%)	43 (58.90%)	0.235
	Single	183 (32.91%)	153 (31.68%)	30 (41.10%)	
	Formal relationship	3 (0.54%)	3 (0.62%)	0 (0.00%)	

n—number of cases, %—percentage of the total study group.

**Table 2:** Descriptive statistics for selected scales with respect to the risk of the compulsive buying disorder.

Selected Scales	Total (n = 556)	Norm (n = 483) Group 1	Risk of Compulsive Buying (n = 73) Group 2	*p*
BDI I-II Me (Q1–Q3)	4.5 (1.0–10.0)	4.0 (1.0–9.0)	8.0 (1.0–15.0)	0.021
Neuroticism acc. to NEO-FFI, Me (Q1–Q3)	21.0 (15.0–28.0)	21.0 (15.0–28.0)	24.0 (20.0–32.0)	0.003
Extraversion acc. to NEO-FFI, Me (Q1–Q3)	29.0 (24.0–34.0)	29.0 (25.0–34.0)	30.0 (24.0–35.0)	0.481
Openness to experience acc. to NEO-FFI, Me (Q1–Q3)	26.0 (23.0–31.0)	26.0 (23.0–31.0)	26.0 (23.0–30.0)	0.774
Agreeableness acc. to NEO-FFI, Me (Q1–Q3)	30.0 (27.0–34.0)	31.0 (27.0–34.0)	27.0 (24.0–32.0)	<0.001
Conscientiousness acc. to NEO-FFI, Me (Q1–Q3)	34.0 (29.0–38.0)	34 (30.0–39.0)	30.0 (25.0–38.0)	0.028
Cognitive Restraint of Eating acc. to TFEQ-13, Me (Q1–Q3)	6.0 (4.0–8.0)	6.0 (4.0–8.0)	7.0 (5.0–9.0)	0.004
Uncontrolled Eating acc. to TFEQ-13, Me (Q1–Q3)	5.5 (4.0–7.0)	5.0 (4.0–7.0)	7.0 (5.0–8.0)	0.019
Emotional Eating acc. to TFEQ-13, Me (Q1–Q3)	4.0 (3.0–5.0)	4.0 (3.0–5.0)	4.0 (3.0–5.0)	0.533
WART, Me (Q1–Q3)	53.0 (45.0–62.0)	51.0 (44.0–61.0)	60.0 (51.0–66.0)	<0.001
Addiction to work acc. to WART, n (%)	79	60 (12.42%)	19 (26.03%)	<0.001

BDI I-II—Beck Depression Inventory, NEO-FFI—Personality Inventory NEO-FFI, TFEQ-13—Three-Factor Eating Questionnaire, WART—Work Addiction Risk Test, Me—median, Q1—quartile first, Q3—quartile third, n—number of patients, *p*—statistical significance.

**Table 3:** Logistic regression for the compulsive buying disorder group.

Selected Scales	Model I		Model II			Model III		
	OR (Cl-95%–CI + 95%)	*p*	OR (Cl-95%–CI + 95%)	*p*	AIC	OR (Cl-95%–CI + 95%)	*p*	AIC
BDI I-II	1.051 (1.021–1.081)	0.001	1.052 (1.022–1.083)	0.001	420.1	1.050 (1.020–1.081)	0.001	427.9
Neuroticism acc. to NEO-FFI	1.040 (1.013–1.067)	0.003	1.034 (1.007–1.061)	0.012	424.5	1.034 (1.007–1.062)	0.014	431.7
Extraversion acc. to NEO-FFI	1.017 (0.982–1.054)	0.351	1.010 (0.975–1.047)	0.577	430.5	1.011 (0.974–1.048)	0.572	438.1
Openness to experience acc. to NEO-FFI	0.997 (0.958–1.038)	0.900	0.993 (0.953–1.034)	0.722	430.7	0.989 (0.950–1.031)	0.612	438.2
Agreeableness acc. to NEO-FFI	0.937 (0.900–0.976)	0.002	0.943 (0.906–0.981)	0.004	422.2	0.942 (0.904–0.981)	0.004	429.7
Conscientiousness acc. to NEO-FFI	0.964 (0.932–0.996)	0.030	0.968 (0.936–1.001)	0.057	427.3	0.969 (0.936–1.002)	0.064	435.1
Cognitive Restraint of Eating acc. to TFEQ-13	1.130 (1.038–1.23)	0.005	1.122 (1.029–1.224)	0.009	424.6	1.123 (1.029–1.226)	0.010	431.8
Uncontrolled Eating acc. to TFEQ-13	1.109 (1.014–1.212)	0.024	1.103 (1.007–1.208)	0.035	426.3	1.100 (1.004–1.206)	0.040	434.3
Emotional Eating acc. to TFEQ-13	0.969 (0.826–1.136)	0.695	0.957 (0.813–1.126)	0.595	430.6	0.959 (0.815–1.129)	0.615	438.2
WART	1.051 (1.03–1.073)	<0.001	1.050 (1.030–1.073)	<0.001	407.4	1.052 (1.030–1.074)	<0.001	414.7
Addiction to work acc. to WART	2.481 (1.377–4.469)	0.002	2.384 (1.318–4.314)	0.004	423.3	2.435 (1.340–4.423)	0.003	430.7

BDI I-II—Beck Depression Inventory, NEO-FFI—Personality Inventory NEO-FFI, TFEQ-13—Three-Factor Eating Questionnaire, WART—Work Addiction Risk Test, OR—odds ratio, CI—confidence interval, *p*—statistical significance. Notes: Model I was univariate regression. Model II was adjusted by age.

## Data Availability

The data presented in this study are available upon request from the first author.
